# Extreme Microstomia in an 8-Month-Old Infant: Bilateral Commissuroplasty Using Rhomboid Buccal Mucosa Flaps

**Published:** 2009-12-26

**Authors:** Patrick Jaminet, Frank Werdin, Armin Kraus, Matthias Pfau, Hans-Eberhard Schaller, Stephan Becker, Nektarios Sinis

**Affiliations:** ^a^ Department of Plastic, Hand and Reconstructive Surgery, Burn Center, BG-Trauma Center, Eberhard-Karl University of Tübingen, Schnarrenbergstr. 95, 72076 Tübingen, Germany; ^b^ Department of Oral and Maxillofacial Surgery, University of Kiel, Arnold-Heller-Str. 16, 24105 Kiel, Germany

## Abstract

**Objective:** A case of extreme microstomia in an 8-month-old infant is presented. As a result of caustic acid ingestion at the age of a few weeks, the male infant developed progressive stricture of the perioral region preventing him from normal food intake. **Methods:** The patient was treated by bilateral commissurotomies and a total of 4 rhomboid flaps based in the buccal mucosa. **Results:** We were able to enlarge the mouth aperture and subsequently cover the created soft tissue defects, with good esthetic result. The patient learned to suck the feeding bottle and was able to demonstrate oral dynamics, including laughing and crying. **Conclusion:** We present our surgical technique, the postoperative functional and esthetic outcome, and a brief literature review. Only few publications deal with the same matter and none with a similar life-threatening case.

During the early 1900s, microstomia was frequently due to accidental electrical burns or ingestion of caustic substances introduced as household cleaners. The subsequent scarring around the mouth affected a large number of children. With the introduction of product safety laws, severe microstomia is actually rarely seen in the western world.

Individuals with microstomia experience several problems: losing the normal function of the lip, eating and drinking, speech and sound production, and forceful blowing and kissing are dramatically limited. Doubtless, the biggest disability is found in case of severe regurgitation where aspiration can induce fatal consequences. Finally, psychological distress is common among affected patients.

## CASE REPORT

An 8-month-old male toddler from Cameroun presented at our outpatient clinic. A German human aid organization (Afrika-Projekte) appealed to us, requesting to consider his poor medical condition. As a newborn twin, he was the victim of a murder assault by the use of acid ingestion. In consequence of subsequent scarring, severe microstomia developed. The infant could be fed only with a syringe dripping fluid into the constricted oral cavity and kept alive.

**Figure 1:**
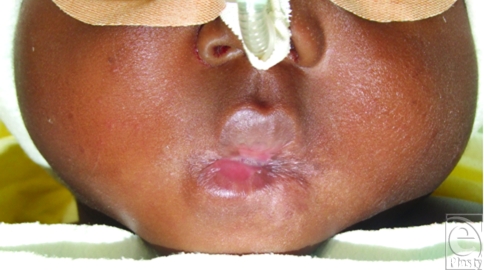
Severe microstomia with a mouth aperture of 2 mm.

On the first physical examination, the patient showed severe microstomia with scar contraction of both commissures. The largest horizontal mouth aperture measured no more than 2 mm (Fig 1). He was breathing mainly through his nose. A preoperative endoscopy of the oral cavity did not reveal any further intraoral scarring and the oesophagus showed healthy mucosa.

**Figure 2:**
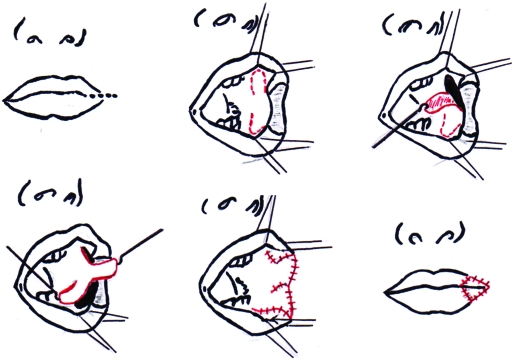
Sequence of operative steps illustrating the elevation of buccal mucosa flaps to cover the soft tissue defects caused by enlarging the oral mouth aperture.

**Figure 3:**
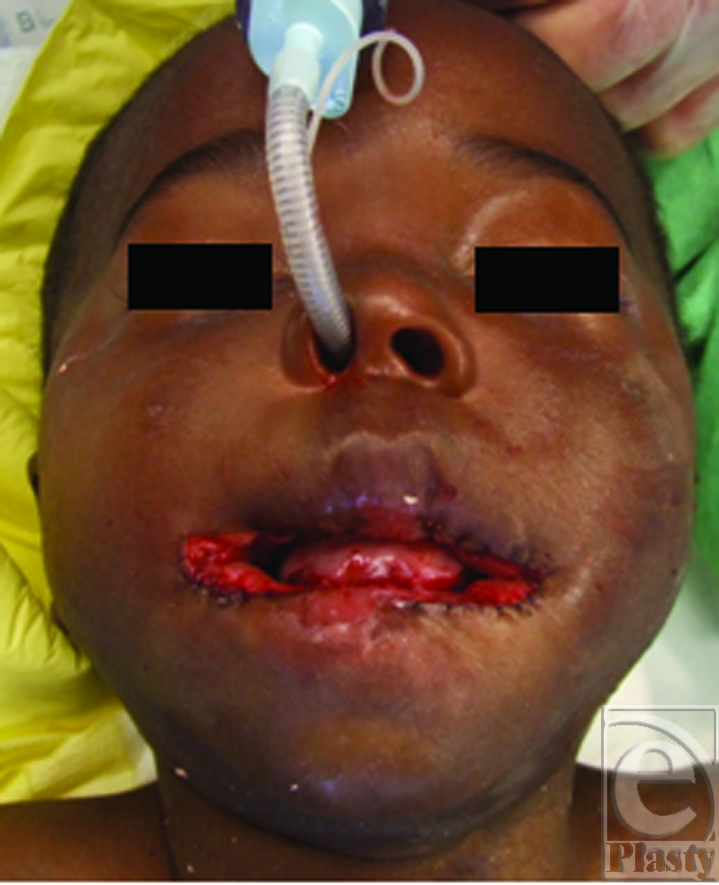
Immediate postoperative aspect with slight overcorrection of the mouth aperture.

**Figure 4:**
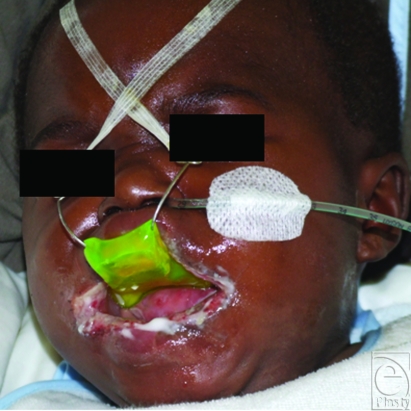
Postoperative palatal splint fixed on the forehead using adhesive tape; nasal gastric tube.

The reconstruction of an adequate mouth opening was performed by enlarging the oral aperture by 2 horizontal cuts of 10 mm each. Then a total of 4 rhomboid buccal flaps were raised including mucosa and submucosa to cover the surgically created defects (Figs 2 and 3). Surprisingly, in spite of the preoperatively performed endoscopy, the bottom of the tongue was attached to the oral diaphragm and had to be released by blunt manipulation. At the end of the operation, a splint was adjusted to the palate and fixed to his forehead by adhesive tape (Fig 4). The harvesting areas of the rhomboid flaps were closed with 6-0 monofil resorbable suture material.

**Figure 5:**
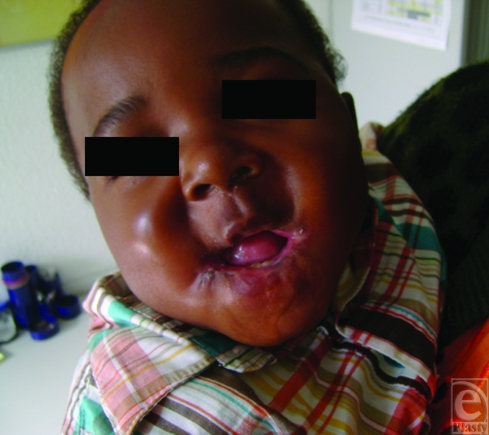
Postoperative result 3 weeks after surgery. The toddler is able to suck the feeding bottle and demonstrates a bright smile.

In postoperative care, the preformed splint prevented the young patient from closing his mouth. The tongue was hourly mechanically mobilized from the oral floor to avoid new scarification. The first 8 days, he received nutrition through a nasal stomach tube. After removing the splint, he rapidly learned suction of the feeding bottle. Two weeks after the operation, the flaps were healed with an acceptable esthetic result (Fig 5). After 3 weeks, the little patient and his mother returned to Cameroun.

The 3-week stay and his medical treatment could not have been achieved without the teamwork of several clinical departments at the University of Tuebingen (Plastic and Reconstructive Surgery, Anesthesiology, Pediatrics, Dentists and Logopedists) and the financial support from both the aid organization and the hospital management mainly of the BG-Trauma Center in Tuebingen.

## DISCUSSION

Restoration of the oral commissure is always a difficult procedure related to the complex functional and esthetic entity of the mouth. Cosmesis and function can be favorably improved depending on which reconstructive technique is chosen. In 1829, the first technique to correct microstomia was presented by Dieffenbach.^1^ It involved the advancement of superior, inferior, and lateral mucosal flaps to reconstruct the corner of the mouth after removal of a triangular wedge of scar tissue. The procedure was modified by Converse^2^ and later by Mehra et al.^3^ After commissurotomy, the named authors preferred either a vermilion advancement or the transposition of the buccal mucosa. Gillies and Millard^4^ presented a technique, using a vermilion flap to reconstruct the upper lip and an oral mucosal advancement flap for the lower lip. Another technique described by Villoria^5^ transposed inner and outer orbicularis oris muscle flaps and advanced oral mucosa to form the new vermilion. Johns et al^6^ reported the use of a triangular pedicled flap for oral commissuroplasty, with good result. Muhlbauer^7^ proposed 2 Z-plasties, using the rotation of 2 small skin flaps into the mucosa of the lip. Fairbanks and Dingman^8^ reconstructed the oral aperture by obliquely dividing the existing vermilion into 2 diminishing flaps approximated to the new mouth angle. Takato et al^9^ used a free forearm flap for reconstruction of the oral cavity and vermilion flaps at the oral commissure on a patient with severely constricted oral cavity because of mucosal adhesions.

In our case, we reconstructed both corners of the mouth, using 4 rhomboid flaps from the buccal mucosa in a technique described by Martins et al.^10^ For our little patient, the esthetic result was of only secondary importance. The primary goal was to allow him food intake for survival and a normal speech development for later social contact. Therefore, after commissurotomy, we reconstructed the new mouth angles by 4 rotating rhomboid buccal flaps to cover the raw surfaces, thus making the procedure as simple as possible. The main problem that presented intraoperatively was the attachment of the bottom of the tongue to the oral diaphragm. Therefore the adhesions were released by blunt dissection till the tongue could be pulled out of the mouth reaching a respectable freedom of motion for future speech production (Fig 4). To prevent early scarring, a custom-made splint was fixed to the palate to keep the mouth open and allowing us to mechanically mobilize the tongue hourly. The postoperative course was free of complications. We were able to assess oral dynamics when the infant was laughing or crying. Furthermore, he was able to suck the feeding bottle, thus moving and training his tongue actively after 1 week. Ten days after the operation, the patient was released from the hospital. The young patient and his mother savored a fortnight charity stay closely observed by plastic surgeons, dentists, and logopedists.

The risk of recurring stricture of the mouth angles, readhesion of the tongue, and the incidence of scar carcinoma is high. At this point, reconstruction of the oral cavity, using a free forearm flap as described by Takato et al,^9^ would be a possible option. However, taking the young age of the patient into account, we decided to do the reconstruction by simple means in order to keep complication rates as low as possible. A later free flap reconstruction is still possible and should be discussed when the infant will reach his third or fourth year of age. At this moment a quick solution was arrogated, which should open the strictured mouth to avoid complications from malnutrition or unexpected emesis.

The development of the young patient will be closely followed by us. In case of any complications or recurrency of the stricture and tongue adhesions, we will bear the expenses for his best possible treatment.
